# Sparrowhawk movement, calling, and presence of dead conspecifics differentially impact blue tit (*Cyanistes caeruleus*) vocal and behavioral mobbing responses

**DOI:** 10.1007/s00265-017-2361-x

**Published:** 2017-08-13

**Authors:** Nora V. Carlson, Helen M. Pargeter, Christopher N. Templeton

**Affiliations:** 1 School of Biology, University of St Andrews, Harold Mitchell Building, St Andrews, Fife, Scotland KY16 9TH UK; 2 Present Address: Departament de Biologia Animal (Vertebrats), Universitat de Barcelona, 08028 Barcelona, Spain; 3 Department of Biology, Pacific University, 2043 College Way, Forest Grove, Oregon, 97116 USA

**Keywords:** Anti-predator behavior, Biorobotics, Blue tit, Mobbing, Risk assessment, Taxidermy model

## Abstract

**Abstract:**

Many animals alter their anti-predator behavior in accordance to the threat level of a predator. While much research has examined variation in mobbing responses to different predators, few studies have investigated how anti-predator behavior is affected by changes in a predator’s own state or behavior. We examined the effect of sparrowhawk (*Accipiter nisus*) behavior on the mobbing response of wild blue tits (*Cyanistes caeruleus*) using robotic taxidermy sparrowhawks. We manipulated whether the simulated predator moved its head, produced vocalizations, or held a taxidermy blue tit in its talons. When any sparrowhawk model was present, blue tits decreased foraging and increased anti-predator behavior and vocalizations. Additionally, each manipulation of the model predator’s state (moving, vocalizing, or the presence of a dead conspecific) impacted different types of blue tit anti-predator behavior and vocalizations. These results indicate that different components of mobbing vary according to the specific state of a given predator—beyond its presence or absence—and suggest that each might play a different role in the overall mobbing response. Last, our results indicate that using more life-like predator stimuli—those featuring simple head movements and audio playback of vocalizations—changes how prey respond to the predator; these ‘robo-raptor’ models provide a powerful tool to provide increased realism in simulated predator encounters without sacrificing experimental control.

**Significance statement:**

Anti-predatory behavior is often modulated by the threat level posed by a particular predator. While much research has tested how different types of predators change prey behavior, few experiments have examined how predator behavior affects anti-predatory responses of prey. By experimentally manipulating robotic predators, we show that blue tits not only respond to the presence of a sparrowhawk, by decreasing feeding and increasing anti-predator behavior and vocalizations, but that they vary specific anti-predator behaviors when encountering differently behaving predators (moving, vocalizing, or those with captured prey), suggesting that prey pay attention to their predators’ state and behavior.

**Electronic supplementary material:**

The online version of this article (doi:10.1007/s00265-017-2361-x) contains supplementary material, which is available to authorized users.

An animal’s ability to avoid predation is an important component of its fitness (Devereux et al. 2005). Failing to recognize a predator can have serious consequences (Edelaar and Wright 2006), and as such, prey need to be able to recognize and respond appropriately to predator threats. Prey are often predated upon by a variety of different predators, all of which may pose different kinds and levels of threat (Manser et al. 2002; Templeton et al. 2005). The level of threat a predator poses can also vary with predator features (e.g., hunger levels; Brown and Schwarzbauer 2001), across seasons (DeGregorio et al. 2014), or even between different times of day (e.g., day and night; Halle 1993). The ability to make subtle distinctions about the immediate threat posed by a predator is therefore highly beneficial to prey animals. Prey responses to predators are often used to understand how species develop predator recognition (McLean et al. 1999; Kullberg and Lind 2002), which features different species use to recognize (Beránková et al. 2014) or categorize predators (Griffin et al. 2001; Tvardíková and Fuchs 2010), and how species warn about potential threats (Leavesley and Magrath 2005; Templeton et al. 2005; Gill and Bierema 2013). As many species may learn about predators through social or personal experience, predator models are frequently used to train naïve individuals to recognize novel threats, both to understand the mechanisms controlling associative learning (Magrath et al. 2015) and in conservation efforts to prepare captive bred individuals in for release in the wild (Maloney and McLean 1995; Griffin et al. 2000). To effectively address either of these questions, it is imperative to first determine what features (morphological, behavioral, or otherwise) prey use to assess the threats posed by a predator. However, few studies have investigated how anti-predator behavior is affected by changes in a predator’s own state or behavior. This study addresses this gap in knowledge by using robotic stimuli (Partan et al. 2010; Frohnwieser et al. 2016) to examine behavioral responses of prey to different predator states. Specifically, we used robotic sparrowhawks (*Accipiter nisus*) to simulate differences in a predator’s behavior and state to determine how these variables affect mobbing responses of blue tits (*Cyanistes caeruleus*), a preferred prey.

Prey can use a variety of different features to assess the relative threat level of predators, with auditory and visual cues being the predominant modalities used in avian systems (Suhonen 1993; Quinn et al. 2006). Birds have well-developed hearing (Dooling and Therrien 2012), and many species recognize the vocalizations of predators (Billings et al. 2015), the sounds predators make moving through their environment (Magrath et al. 2007), and the warning signals of conspecifics or heterospecifics (Templeton and Greene 2007). Auditory signals are so important that species will increase vigilance behavior if the acoustic environment is negatively impacted by noise (Quinn et al. 2006). Visual cues tend to travel over shorter distances than auditory cues and provide a relatively narrow field of detection (Stevens 2013), but are also important in recognizing and categorizing predators. Animals assess predator threat using a variety of visual cues, ranging from a predator’s body shape (Cook et al. 2013), beak and eye shape (Beránková et al. 2014), coloration (Davies and Welbergen 2008), and texture (Němec et al. 2014). For example, Němec et al. (2014) found that nesting red-backed shrikes (*Lanius collurio*) mob Eurasian jay (*Carrusul glandarius*) dummies when placed near their nest, but they responded most strongly to stuffed taxidermy jays, less strongly to plush toy jays, and least strongly to silicone dummies. Visual cues can often provide more detailed information about predators, and prey will commonly approach and inspect an auditory source of information to acquire further visual information about a predator (Nocera et al. 2008). In addition to physical features of a predator, prey can assess a predator’s current state (e.g., hunger level; Brown and Schwarzbauer 2001), in order to determine the threat level of a given predator encounter. Some birds can assess a predator’s speed of approach (Bateman and Fleming 2011), distance (Stankowich and Blumstein 2005), attention (Clucas et al. 2013; Book and Freeberg 2015), behavior (Griesser 2008), whether a predator is migrating (Edelaar and Wright 2006), and whether it is perched or flying (Gill and Bierema 2013) as means to estimate whether it is currently hunting. Assessing a predator’s state could allow a prey animal to make more subtle judgments about risk, which could be important in reducing the overall costs of anti-predator behavior (Cresswell 2008).

Research examining the type and amount of information prey extract from encounters with predators has employed a variety of different predator stimuli. Some studies have used live predators (e.g., Templeton et al. 2005) to provide the most realistic experimental conditions. However, using live predators is often not feasible for ethical, practical, or experimental reasons (e.g., Tvardíková and Fuchs 2010). Researchers have used a variety of predator models, including those made from wood (Bartmess-LeVasseur et al. 2010; Beránková et al. 2014; Němec et al. 2014), plastic (Conover 1985), fabric (Němec et al. 2014; Book and Freeberg 2015), or taxidermy mounts or study skins of real predators (Curio 1978; Suzuki 2014). Regardless of the type of predator model used, most studies, though not all (Conover 1985), have presented the models statically, with the predator remaining completely stationary and quiet throughout the simulated encounter. While static models are often successful in eliciting mobbing or fleeing responses from target species, the limitations of using models, the similarity of the responses they elicit to live predators, and the effects of predator behavior are often ignored (but see: Conover 1985; Chandler and Rose 1988). If and how predator behavior or state affects the anti-predator responses of their prey remains poorly understood as studies that have included predator behavior or state often use different model materials which can impact the anti-predator response (Conover 1985; Chandler and Rose 1988; Němec et al. 2014).

To determine how a predator’s behavior and state affect its prey’s anti-predator response, we tested how blue tits behave in response to robotic taxidermy sparrowhawk models that exhibited different behaviors and states. Blue tits are common Eurasian songbirds, and sparrowhawks are their commonest high-threat avian predator. We presented wintering flocks of blue tits with sparrowhawk models that varied in three different behaviors (calling or moving) and states (caught prey or not). We predicted that all sparrowhawk models would elicit a heightened mobbing response from blue tits, but also that the specific behavior and state of the sparrowhawk would impact the tits mobbing behavior. Specifically, we predicted that both calling and moving sparrowhawks would elicit a heightened mobbing response from blue tits compared with stationary and silent sparrowhawks (i.e., increases in scanning, wing-flicking, and call rate and decreases in feeding), as calls would allow individuals to quickly locate and respond to predators and head movement would make avoiding predator attention (i.e., gaze) more difficult. We predicted that predators with a dead conspecific in their talons would both decrease the intensity of the mobbing response (i.e., decreased wing-flicking and call rate and increased feeding) as it should indicate a lesser threat because the predator has caught something and unlikely to hunt again immediately; the presence of a dead conspecific could also increase investigatory behavior (i.e., scanning) as the dead tit may supply more information about the predator’s state and hunting capabilities.

## Methods

### Subjects and study sites

We chose blue tits, a small passerine species often found in mixed species flocks in the winter (Perrins 1979), as our study species. One of the main predators of adult, fledged, and juvenile blue tits is the sparrowhawk (Perrins 1979). Sparrowhawks pose a particular threat as they are effective at catching small birds (Dial et al. 2008) and have a diet composed mostly of small birds (Zawadzka and Zawadzki 2001). One defense mechanism tit species employ when confronted with a sparrowhawk is mobbing, a behavior that serves to harass and drive off a predator that is perched (Morse 1973). Mobbing behavior includes a combination of stereotyped agitation behavior, such as flicking the wings out and scanning for predators, which are typically combined with mobbing call production (Curio 1975, 1978). Blue tits are aggressive mobbers (Randler and Vollmer 2013), are common in our study population, and respond to sparrowhawks by aggressively mobbing them (Carlson et al. 2017a).

**Fig. 1:**
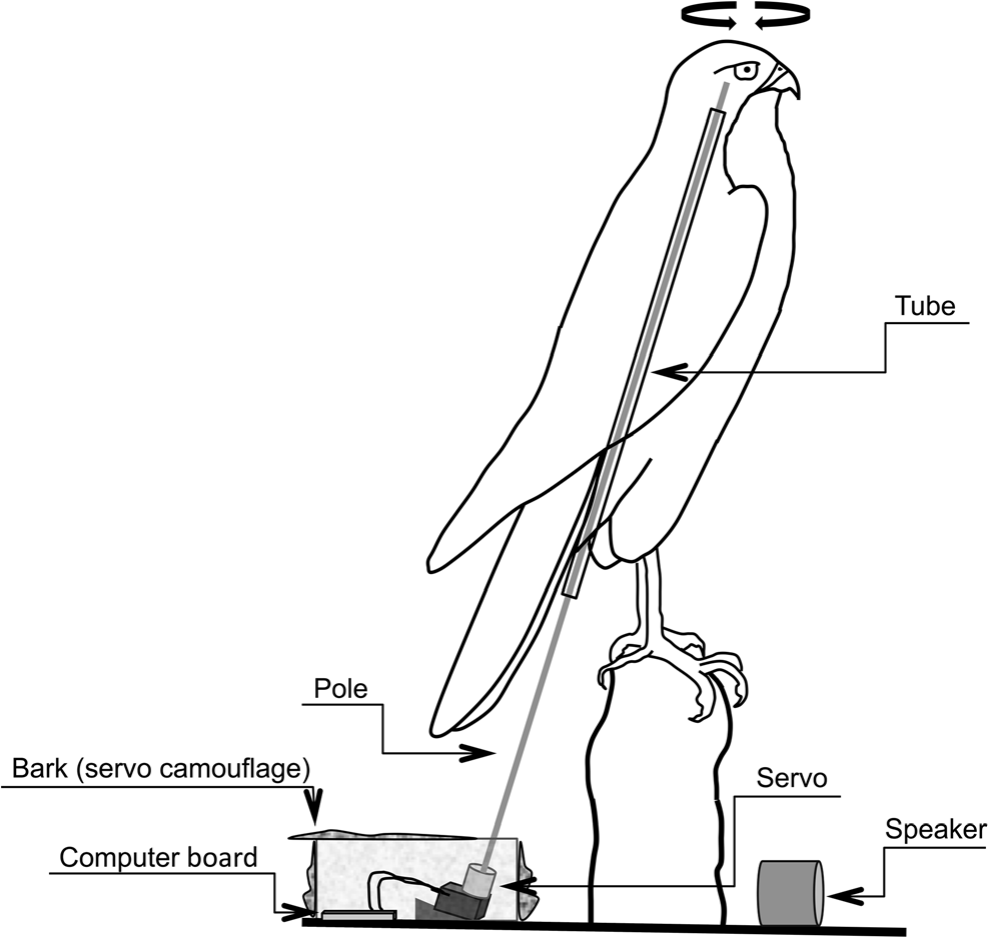
Schematic of the robo-sparrowhawk used for these experiments

The study was carried out in winter (January–February 2015) when tit flocks are most prevalent and birds regularly visit feeders. Study sites were bird feeders located in private and public gardens within the town of St Andrews, Scotland, UK (56.340° N, 2.796° W; Fig. 1). Blue tits readily use feeders during the winter and quickly habituate to human presence around these feeders. We used a total of 14 study sites: 12 sites where we completed all treatments and 2 supplementary sites where we conducted 3 trials that could not be completed at two of the initial sites due to the arrival and continued presence of a real sparrowhawk part way through the experiment. All sites were situated at least 400 m away from one another in order to ensure that they were independent as at this distance, it is unlikely to find a tit from a neighboring flock (Hinde 1952; Ekman 1979; Cramp 1993; Bartmess-LeVasseur et al. 2010).

### Stimuli

We examined the response of tits to taxidermy sparrowhawk mounts varying in several different key features. A total of five treatments were used in the study, which varied three features of the sparrowhawk’s behavior that tits might key in on when identifying predators: movement, calling, and presence of captured prey. We chose to include moving (movement of the sparrowhawk’s head) and calling as visual and auditory cues are commonly used by birds to assess the threat posed by predators. We included caught prey as this not only suggests that the predator maybe satiated and will be less likely to continue hunting, but also due to recent research showing that individuals are attracted to dead conspecifics (Iglesias et al. 2014; Swift and Marzluff 2015), presumably as a means to learn about dangerous situations (Curio et al. 1978a; Conover and Perito 1981). We used taxidermy sparrowhawk mounts to generate five different treatments combining these variables: (1) positive control: still and silent model; (2) captured prey: still and silent model with a captured blue tit (also a model) in its talons, henceforth referred to as ‘dead-tit’; (3) calling only: still model with calling sparrowhawk playback, (4) moving only: moving model that was silent; and (5) combined moving and calling: moving and calling model. We describe how we manipulated each of these variables below.

#### Movement: robotic raptors

We tested the effect of predator head movement by using robotic sparrowhawks (Online Resource 1). We constructed these robots by either including the moving parts during the taxidermy process (sparrowhawk a) or by taking the head off of the bird post-taxidermy, fitting the robotics, and re-assembling (sparrowhawk b). To construct the robo-raptors, we put a hollow tube through the body along the natural plane of head movements. Inside the tube, we put a pole that was attached (using either U-POL™ body filler from U-POL, London, UK) or UHU© all-purpose adhesive glue (GmbH & Co. KG, Bühl/Baden, Germany) to the inside of the bird’s skull on one end and to a servo motor (Futaba S3003 from Futaba Corporation Oshiba, Japan or Hitec HS-422 Delux from Hitec RCD, Poway, CA, USA), using a 5/32 in. servo shaft coupler (Futaba or Hitec respectively). We controlled the rotation of the head (via the servo) with an Arduino computer (Arduino Duemilanove from Arduino LLC, https://www.arduino.cc) and 9v battery pack (Fig. 1). We wrote a simple computer program composed of a loop of a series of 15 different movements where the head turned between 2 and 110°. Degree changes and movement delay times were based on natural movements of videotaped accipiters (E. Greene, pers. com.). The rotation of the head mimicked natural sparrowhawk behavior and did not exceed the natural rotational degree of a live sparrowhawk (Online Resource 1). The electronics were hidden in a small box under the raptor’s perch and concealed by bark and lichens. We used two different taxidermy sparrowhawk mounts, one juvenile male and one adult female, to reduce pseudoreplication, and the same mount was used for each trial (with the motor switched on for movement trials and off for still trials) at a particular study site to remove any biases between the two models.


ESM 1(MP4 29,473 kb)


#### Vocalizations

Although sparrowhawks are silent ambush predators, they often attract the attention of conspecifics by calling (Newton 1986). To test if sparrowhawk vocalizations affected blue tit mobbing behavior, we manipulated whether audio recordings accompanied the robo-raptor during trials. We made playback files of sparrowhawk vocalizations from vocalizations obtained from xeno-canto (http://www.xeno-canto.org) and we chose only calls with high signal to noise ratio and no background noises or other species calling. We then used Raven Pro 1.5 (Bioacoustics Research Program, The Cornell Lab of Ornithology, Ithaca NY) to create 4 different (24 bit, 48 kHz, WAV files) playback exemplars. Each recording contained 8 ‘kekeke’ calls and lasted for 1 min. In order to include much of the variation in commonly produced sparrowhawk calls, each playback contained 4 fast kekeke calls (mean ± standard error; rate: 5.8 ± 0.12 notes/s; length: 0.04 ± 0.003 s; peak frequency: 3.2 ± 0.5 kHz) and 4 slow kekeke calls (rate: 1.2 ± 0.04 notes/s; length: 0.25 ± 0.008 s; peak frequency: 3.7 ± 0.5 kHz) which were separated by an inter-call interval of 3.8 ± 0.06 s. The order of these calls was randomized in each recording. We used four different exemplars to reduce pseudoreplication. During the trials, the calls were played from a SanDisk Sansa Clip + Player (SanDisk Corporation, Milpitas, CA, USA) on a X-mini II Capsule speaker (Xmi Pte Ltd., Singapore; frequency response: 100 Hz–20 kHz) attached to the base of the sparrowhawk mount. Calls were played at natural amplitude of approximately 80 dB (SPL at 1 m re: 20 μPa).

#### Captured prey

To simulate a sparrowhawk that had recently captured conspecific prey, we placed a taxidermy blue tit sideways in the talons of the sparrowhawk model. We used two different taxidermy blue tits as the ‘prey’ in the captured prey trials to reduce pseudoreplication. Both specimens were of unknown sex and relatively disheveled, resulting in a realistic simulation of a recently captured tit. Captured prey trials were always conducted with the sparrowhawk silent and still.

## Experimental procedure: presentation of stimuli

A total of 60 trials were completed, 12 of each of the 5 stimuli. We used a repeated measures design, conducting each treatment at each of the sites (with the exception of 2 sites previously described). As we could not identify individual birds, we treated each location as an independent sampling unit. We left a minimum of 2 days between trials at each site to avoid habituation, with the average period between trials being 5.57 days. In order to control for temporal effects and eliminate any effects of priming (Němec et al. 2014), we presented stimuli in a different order at each site and balanced them such that there was an equal number of each stimuli across the 1st to 5th trial. The specific mount, audio recording, and dead tit were randomly allocated to each site, and the same exemplar of each was used for all trials at a given site. Blue tit flock size varied across treatments (mean ± SE: 1.6 ± 0.05 birds; range: 1–5 birds). Other species present included the following: great tits *Parus major* (mean: 0.44 ± 0.07; range = 0–4 birds) and coal tits, *Pariparus ater* (mean: 0.28 ± 0.06 birds, range: 0–5 birds),

Each location had a feeder present which was stocked from about 2 months prior to the experiment start date with black oil sunflower seed. All feeders were seed feeders, and while some locations had other feeders present, they were in a separate part of the property from the feeder presentations that were conducted at, and appeared to have no effect on flock composition or responses to the experimental treatment.

Prior to the trial, we placed the presentation stand (a 1.5-m high pole topped with a small wooden platform) approximately 2 m from the feeder and adjacent to several good natural perches, such as the branch of a tree or fence post a minimum of 1 m from cover. Trials began when the experimenter visually identified at least one blue tit present within 5 m from the sparrowhawk mount stand. The observer then recorded a 3-min pre-trial period to establish baseline behavior in the absence of any predators before each treatment. To begin the predator presentation trial, we carried the sparrowhawk mount uncovered and placed it on the stand facing towards the feeder; if the trial was a moving, calling, or with a dead tit stimuli, the predator would begin those ‘actions’ as soon as it was on the stand. After placing the predator near the feeder, the experimenter returned to cover (a minimum of 8 m from the sparrowhawk) and began recording. All recordings were annotated by the observer and included information about the behavior of the blue tits, the number of blue tits in the area, and the number of great tits and/or coal tits present. Recordings continued for 30 min after the sparrowhawk was presented.

We recorded four different blue tit behaviors for all trials: foraging (either a successful visits to the feeder or pecking or manipulating other food items with their beak away from the feeder), scanning (obvious head movements where the bird looked up or from side to side), wing-flicks (flicking their wings open then closed without taking off), and calling (producing any mobbing vocalizations; Carlson et al. 2017a). All behaviors are mutually exclusive with one another except for calling which can occur with any behavior. We chose these behaviors for three reasons: (1) each of these behaviors change in response to increase in stress or perceived danger and are used as indicators of stress or perceived danger in many species of birds (Andrew 1956; Curio et al. 1978b); (2) each of these behaviors are common during mobbing events, with the exception of foraging which is common outside of mobbing events (Curio et al. 1978b); (3) each behavior is driven by different motivations: feeding is a non-stress behavior driven by hunger, scanning is an individual stress and/or investigative behavior driven by increased perception of danger and the need for more information, and wing-flicking is a social high-stress behavior driven by high levels of perceived threat. In addition to mobbing behaviors, we also examined variation in call rate across trials, as blue tits, like other Paridae, change their call rate in response to the degree of threat a predator poses (Templeton et al. 2005; Carlson et al. 2017a).

Although recordings lasted for 30 min after the sparrowhawk was exposed, mobbing events in tits only last about 2–5 min before the tits present either leave or resume foraging (NVC, HMP, CNT personal observation). Therefore, we decided to analyze only the first 3 min of data after the first mobbing event was initiated. This period began when an individual was oriented towards the sparrowhawk and came within 5 m of the model. As behavior was recorded as it happened, which could potentially have introduced reporting bias for repeated behaviors, we chose to standardize the behavior reporting. To do this, we broke each 3-min section (both pre-presentation and presentation) into 30-s blocks, and for each 30-s block, we marked each physical behavior as either present (any individual blue tit exhibited the behavior) or absent (no individual exhibited the behavior). We then calculated behavior ‘rates’ by summing the total number of 30-s blocks during the 3-min trial that a behavior was exhibited and dividing that number by the total number of 30-s blocks (e.g., blocks where feeding was present/total number of blocks). We calculated call rates by counting the total number of calls produced during the coinciding 3-min mobbing response and divided that total by the number of individuals present. Due to losing sight of birds occasionally, or birds leaving before the 3 min were up, some trials did not have six 30-s blocks (mean ± SE; 5.14 ± 0.12). Overall flock size varied somewhat across trials and treatments (mean ± std. error: still-silent 1.46 ± 0.16, dead tit 1.84 ± 0.16, moving-silent 1.71 ± 0.19, still-calling 1.81 ± 0.19, moving-calling 1.60 ± 0.29 blue tits), so we accounted for this by including a random flock size term in the statistical models. All behavioral observations were conducted by the same individual (HMP) to remove between observer variation. Due to the nature of the data collection in the field, the observer was not blind to treatment.

Although these behaviors and vocalizations are all components of a mobbing response, only two response variables were significantly correlated (call rate and scanning: Pearson’s *r* = −0.34, *P* = 0.014; all other paired correlations *P* > 0.05). Data reduction techniques were therefore uninformative, with principle components analysis resulting in each behavior primarily loading on its own component. Because we were interested in whether there are fine-scale differences in mobbing behavior in response to differences in predator behavior and state, we analyzed each response variable separately. By analyzing the behaviors separately, we could examine whether different predator behaviors elicited different types of behavioral responses from blue tits, thereby providing more detailed information about the differences in blue tit perception and responses to predators with different predatory behaviors/states.

All trials were recorded using a Marantz PMD660 solid-state sound recorder (Marantz America, LLC., Mahwah, N.J., USA) at a sampling rate of 44.1 kHz and Sennheiser ME 66 super-cardioid microphone (Sennheiser Electronics, Hanover, Germany) from a distance of approximately 8 m. All trials began at least an hour after sunrise and finished at least an hour before sunset to reduce stress on the birds while they recovered/prepared the overnight period and eliminate confounding effects of low light levels on predator detection and response (Rodríguez et al. 2001). Time of day was not included in the analysis.

## Statistical analysis

To test whether sparrowhawk movement, vocalizations, or the presence of a dead tit affected blue tit behavior, we generated linear mixed models using lme4 statistical package (Bates et al. 2014) in R (R Core Team 2014). We first tested whether blue tits responded to the presence of a sparrowhawk by increasing mobbing-related behaviors after the sparrowhawk was presented compared to pre-presentation across all sparrowhawk treatments. To do this, we generated linear mixed models with Gaussian distribution and an identity link function and included treatment (pre-presentation negative control, still-silent positive control, dead tit, moving-silent, still-calling, and moving-calling) as our response variable. We included sparrowhawk exemplar and trial order as fixed effects, and average number of blue tits present, average number of great tits present, average number of coal tits present, and feeder location random effects.

To test whether sparrowhawk treatment had a significant effect on blue tit behavior, we ran type III Wald Chi-square tests on the model and took the Bonferroni adjusted α value of *α* = 0.013 as our limit for the type III Wald Chi-square tests. We ran planned comparisons by setting the positive control to the intercept to determine if the sparrowhawk’s behavior or state affected blue tit behavioral response. Our models were fit using REML and *t* tests used Satterthwaite approximations to estimate degrees of freedom as this is one accepted method for estimating degrees of freedom for mixed models in order to generate *p* values (Witkovský 2012). We did not correct these planned comparisons for multiple tests as it can be argued that as they were orthogonal (all treatments are tested against the negative control), no experiment-wise type I error rate corrections are necessary (Ruxton and Beauchamp 2008) and using a method such as a Bonferroni correction could be overly stringent and increase the chance of committing type II errors to the point that we may overlook important differences in blue tit behavior (Rothman 1990; Perneger 1998; Feise 2002).

## Results

### Effects of sparrowhawk presence

**Fig. 2:**
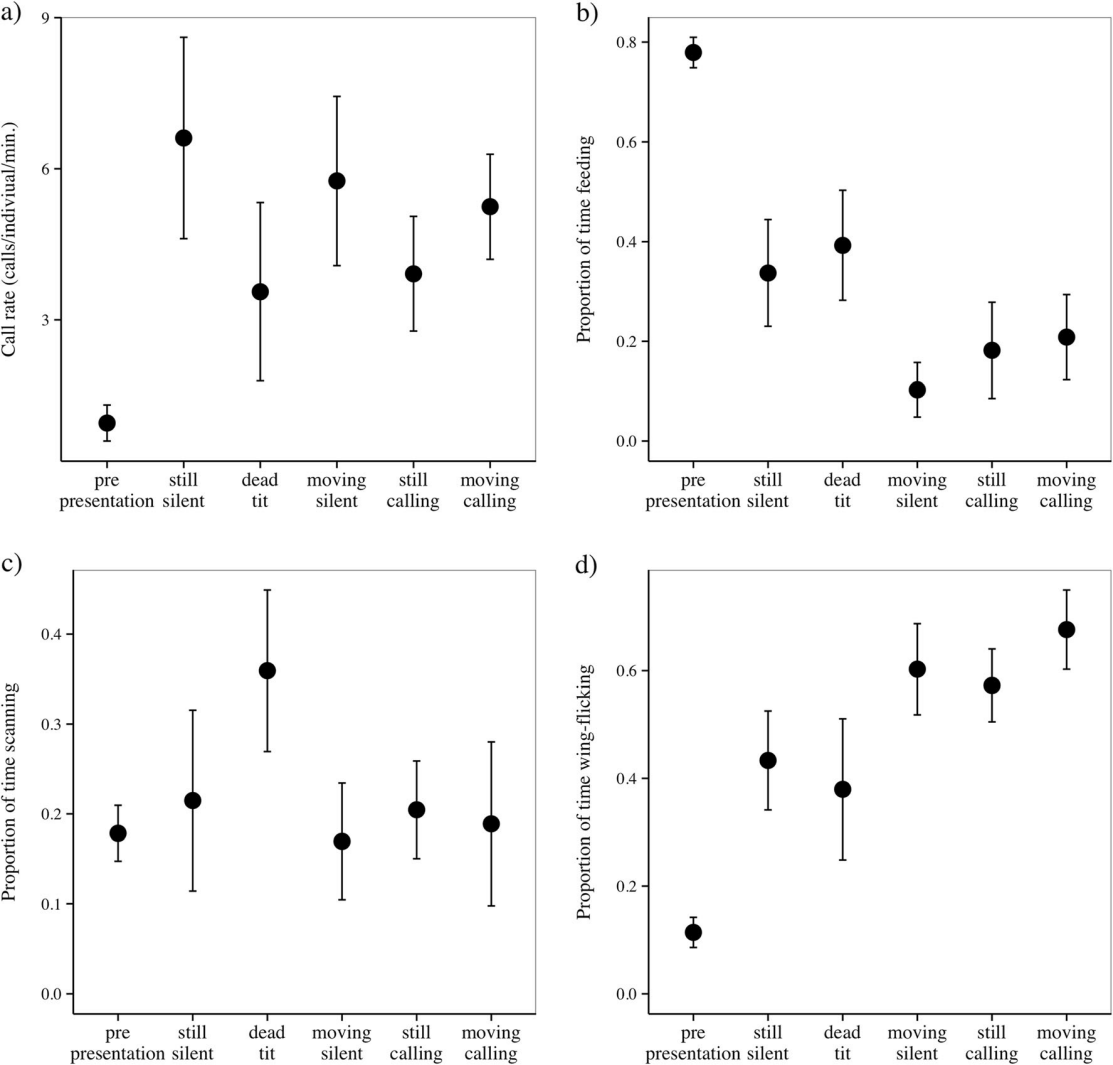
Blue tit mean (± standard error) **a** calling, **b** feeding, **c** scanning, and **d** wing-flicking rates in response to different behavior of sparrowhawk mounts (pre-trial: no mount, still-silent: control silent still mount, dead tit (still silent mount with a dead tit in its talons), moving-silent, still-calling, and moving-calling)

The presence of sparrowhawks affected blue tit behavior, with blue tits responding to the presence of sparrowhawks by decreasing feeding and increasing alarm calling and wing-flicking rates compared with pre-trial periods (Table 1; Fig. 2). In contrast, sparrowhawk presence did not consistently affect the scanning rates of blue tits (Table 1; Fig. 2).

**Table 1:** Linear mixed model type III Wald Chi-square results for predator treatment (pre-playback negative control, still silent positive control, dead tit, moving silent, still calling, moving calling) as a significant predictor of variation in blue tit mobbing response, and planned comparison *t* test results for predator presence and behavior differentiation

Behavior	LMM			Planned comparisons										
				Intercept	Still silent		Dead tit		Moving silent		Still calling		Moving calling	
		*χ* ^*2*^	*P*		*T*	*P*	*T*	*P*	*T*	*P*	*T*	*P*	*T*	*P*
Rate	Stimulus	39.20	*< 0.001*	Pre-playback	4.23	*< 0.001*	2.48	*0.015*	4.06	*< 0.001*	2.05	*0.044*	3.85	*< 0.001*
	Mount	0.06	0.807	still silent	–	–	− 1.33	0.188	− 0.53	0.598	− 1.82	0.073	− 0.70	0.485
	Order	6.18	0.289											
Feeding	Stimulus	139.38	*< 0.001*	Pre-playback	− 4.83	*< 0.001*	− 4.31	*< 0.001*	− 8.24	*< 0.001*	− 7.38	*< 0.001*	− 7.17	*< 0.001*
	Mount	0.00	0.973	still silent	–	–	0.32	0.751	− 2.00	*0.049*	− 1.56	0.122	− 1.23	0.222
	Order	8.62	0.125											
Scanning	Stimulus	2.72	0.743	Pre-playback	0.18	0.856	1.54	0.127	0.54	0.593	0.72	0.471	0.20	0.840
	Mount	5.04	*0.025*	still silent	–	–	1.03	0.305	0.23	0.816	0.38	0.705	0.00	0.997
	Order	22.04	*0.001*											
Wing-flicking	Stimulus	130.48	*< 0.001*	Pre-playback	4.00	*< 0.001*	2.97	*0.004*	7.62	*< 0.001*	5.73	*< 0.001*	7.76	*< 0.001*
	Mount	5.87	*0.015*	still silent	–	–	− 0.58	0.567	2.32	*0.023*	1.32	0.192	2.40	*0.020*
	Order	4.33	0.503											

Bonferroni adjusted α-value for 4 type III Wald Chi-square tests are *α* = 0.013. Italic values indicate *P* values generated from *X* ^*2*^ test statistic *P* ≤ 0.05, *P* values generated from *t* test statistic ≤ 0.05

### Effects of sparrowhawk movement

Blue tits had lower feeding rates in response to moving-silent mounts and higher wing-flicking rates in response to both moving mounts compared to the silent-still control mounts (Table 1; Fig. 2). They did not change any other behavior in response to a moving sparrowhawk mount (Table 1; Fig. 2).

### Effect of sparrowhawk vocalizations

Blue tits had higher wing-flicking rates in response to calling sparrowhawk mounts, compared to silent-still controls, but this difference was only statistically different when the mount also moved (Table 1; Fig. 2). A nonsignificant trend also existed for decreased feeding rates when blue tits encountered calling sparrowhawks (Table 1; Fig. 2). No other behavior changed in response to a calling sparrowhawk mount (Table 1; Fig. 2).

### Effects of sparrowhawk state

No statistical differences existed between blue tit behavior in response to a sparrowhawk with a dead tit compared to the silent-still control (Table 1), but there was a nonsignificant trend for increased scanning in the presence of a dead conspecific (Fig. 2).

### Order and mount effects

Scanning and wing-flicking behaviors were slightly higher in response to the adult female sparrowhawk compared to the juvenile male sparrowhawk (Table 1). However, as each location received only one mount, the mount effects could also reflect response differences between locations. Birds also tended to scan more on the third trial than other trials (Table 1). We saw no other order or mount effects (Table 1).

## Discussion

Sparrowhawk behavior and state, including movement, vocalizations, and the presence of a dead conspecific, had a significant impact on the anti-predator behavior of blue tits. While each of these predator features affected prey response, each seemed to impact blue tit anti-predator behavior in different ways.

## Effects of sparrowhawk presence

As expected, blue tits responded to the presence of a sparrowhawk mount by increasing their calling and wing-flicking rates and decreasing their feeding rates. Both the increase in calling and wing-flicking rates, as well as the decrease in feeding behavior, are indicative of the presence of a perceived threat (Hinde 1954; Carlson et al. 2017a; Carlson et al. 2017b). However, unexpectedly, blue tits did not consistently increase their scanning rates in response to the presence of a sparrowhawk. This was contrary to expectations as vigilance (i.e., scanning behavior) often increases in situations of higher predator threat (Lendrem 1983; Creel et al. 2014). Birds are especially vulnerable when feeding from artificial feeders, like those used in this study, due to the general lack of cover or good sightlines, and this perceived risk could have resulted in elevated levels of scanning during all of the trials; thus, failure to detect statistical differences could be due to a ceiling effect under these experimental conditions.

## Effects of sparrowhawk movement

When they encountered a moving (and silent) sparrowhawk, blue tits decreased their feeding rates compared to the non-moving (and silent) control model. This decrease in foraging behavior may be in direct response to the movement of the sparrowhawk, as predator head movement may increase the perceived threat of a predator because each individual is more likely to find itself in line with the predators gaze just by chance, a situation many species consider higher threat (Carter et al. 2008; Bateman and Fleming 2011; Book and Freeberg 2015). Aside from altering their foraging behavior, blue tits increased their wing-flicking behavior in response to both moving predator mounts. Wing-flicking behavior is considered a flight intention behavior (Daanje 1951), but as it often occurs separate from other behaviors found in genuine takeoff sequences, it is likely that it signals a bird’s readiness to fly or the conflicting drives between approaching and flight (Horwich 1965; Earls 2000). This suggests that blue tits perceive moving predators as a signal of increased threat, possibly because direct or tracking predator gaze appears to illicit stronger fear responses than a predator that is oriented away or not looking directly at an individual (Scaife 1976a, b). If the movement of the head either puts individuals in line of the gaze, mimics prey tracking, or simply suggests a hunting predator, this could explain the heightened preparedness for blue tits to escape and therefore exhibit increased wing-flicking behavior.

## Effects of sparrowhawk vocalizations

There is a trend for calling sparrowhawks to decrease blue tit foraging and increase wing flicking, similar to the response observed to moving sparrowhawks. However, a calling sparrowhawk might actually pose less of a threat than a silent sparrowhawk. The higher similarity in blue tit foraging rates in response to moving calling and still calling compared to the moving calling and moving silent suggests that blue tits may feel less threatened by calling sparrowhawks compared to moving silent ones. This lower perceived threat could be a result of a number of factors. First, sparrowhawks rarely call when actively hunting as they primarily ambush predators, so calling might indicate that they are not actively hunting and therefore pose a lesser threat (Newton 1986). Second, predator vocalizations provide blue tits with information allowing them to use acoustic cues to keep track of the sparrowhawk. When foraging, a blue tit’s head is focused downward, meaning that individuals cannot engage in visual vigilance behaviors that allow them to assess potential threats (Lima and Bednekoff 1999). Many birds rely on both visual and auditory vigilance and combine listening for predator vocalizations with scanning for the sight of predators (Alatalo and Helle 1990; Quinn et al. 2006). When background noise increases masking of important predator sounds, birds will increase their scanning rates (Quinn et al. 2006). The vocalizations from the predators, then, may allow blue tits to increase foraging rates in situations when perched predators are present as they can continue to determine the position of the predator using auditory cues without having to increase scanning behavior. Blue tit wing-flicking behavior also follows this pattern. While moving silent, still calling, and moving calling all result in higher wing-flicking rates, still-calling shows a slightly lower response.

## Effects of multimodal sparrowhawk cues

Contrary to expectations, multimodal cues (moving and calling) did not result in an increased anti-predator behavioral response in blue tits. Rather it appears that blue tits respond to the moving calling sparrowhawk either similarly to the moving-silent or the still-calling mount depending on which specific behavioral response is examined. The fact that blue tits appear to group the moving calling mount with either moving or calling sparrowhawk mounts suggests that blue tits view these two behaviors as having different potential threats. Additionally, the fact that the moving calling sparrowhawk mount is grouped differently depending on which blue tit behavior is analyzed suggests that each of the component mobbing behaviors exhibited by blue tits are driven by separate underlying motivations, each affected by different aspects of a predator’s behavior.

## Effects of captured prey

Blue tits responded similarly to the predator with a captured blue tit and the control still-silent sparrowhawk mount; however, there was a trend for individuals to increase their scanning rates in response to sparrowhawks with captured prey compared to all other sparrowhawk mounts. Scanning is an investigatory behavior, and scanning often increases in situations of heightened threat (Huang et al. 2012). Increased scanning could help blue tits keep track of when the sparrowhawk is done feeding (thus becoming higher threat), could allow them to visually assess the identity of the captured individual (Andersson et al. 1998), or help reinforce the danger level of each predator (Curio 1978). Corvids are known to gain information from observing ‘funerals’ of dead conspecifics (Iglesias et al. 2012, 2014; Swift and Marzluff 2015), and the fact that blue tits appeared to pay particularly close attention to the sparrowhawk mount during the dead conspecific trials suggests that they might also be gleaning important information from these interactions. Blue tits treating the sparrowhawk with a dead conspecific more similarly to the still-silent control mount than the calling or moving mounts suggests that a sparrowhawk that has already captured prey may be treated as less of an immediate threat.

## General conclusions

Whether or not a sparrowhawk model was moving, calling, or had captured prey strongly affected the behavior of blue tits in this study but each affected different types of anti-predator behavior. The fact that these three factors had different effects suggests that each has a somewhat different role in predator assessment and each behavior may be representative of different underlying drives (i.e., investigation vs. escape). These results indicate that blue tits perceive differences in the state of predators and adjust their own behavior accordingly. Our findings suggest that when using model predators to examine prey responses, it is important to take predator behavior into account as these differences could impact threat perception and behavioral measures of mobbing response. Robotic taxidermy models, such as we have employed in this study, provide a simple yet powerful method of increasing predator realism for future experiments.
